# Clinical and demographic characteristics of elderly patients with dementia assisted at an outpatient clinic in Southern Brazil

**DOI:** 10.1590/S1980-57642010DN40100007

**Published:** 2010

**Authors:** Cláudia Godinho, Iulek Gorczevski, Andréa Heisler, Maria Otília Cerveira, Márcia Lorena Chaves

**Affiliations:** 1 Dementia Clinic, Neurology Service, Hospital de Clínicas de Porto Alegre.; 2 Medical Sciences Post-Graduation Course, UFRGS School of Medicine.; 3 Internal Medicine Department, UFRGS School of Medicine.

**Keywords:** tertiary outpatient clinic, dementia, Alzheimer’s disease, vascular dementia, Brazil

## Abstract

The aging of the population is a worldwide phenomenon, where 60% of elders live in developing areas of the world such as Brazil, regions in which few studies have been carried out.

**Objectives:**

The goal of this study was to evaluate the clinical and demographic profile of patients with dementing disorders seen at a specialized outpatient clinic in South Brazil.

**Methods:**

A sample of 105 demented patients seen at the Dementia Outpatient Clinic from Hospital de Clínicas de Porto Alegre (HCPA), Brazil between June 2004 and June 2008. Evaluation of patients consisted of medical history, cognitive testing, assessment of functional status (Activities of Daily Living Scale - ADL; Instrumental Activities Daily Living - IADL) and application of the Neuropsychiatry Inventory (NPI) for behavioral symptoms. Severity of dementia was evaluated based on the CDR scale. All patients underwent laboratory screening tests and brain imaging exams to define etiology of dementia.

**Results:**

Of the whole sample, 71% were female. Age was 79±8 years (mean±SD). Educational level was 4±3 years (mean±SD). Sixty-four patients (60%) presented the diagnosis of Alzheimer’s disease. Of the whole sample, 26.7% were classified as CDR=1, 44% as CDR=2 and 29. 3% as CDR=3. A significant difference on the Mini Mental State Examination (MMSE) and functional status scores was observed among the CDR categories (severity). No significant association was found between severity and impairment on memory tests and behavioral symptoms.

**Conclusions:**

Alzheimer’s disease was the most common etiology, followed by vascular dementia. At diagnosis, most patients presented mild to moderate severity of dementia, independent of cause.

## Abstract (pt)

O envelhecimento da população é um fenômeno mundial, 60% destas pessoas moram em áreas em desenvolvimento do mundo, como no Brasil, onde poucos estudos têm sido conduzidos.

**Objetivos:**

O objetivo deste estudo é avaliar o perfil clínico e demográfico de pacientes com demência vistos em uma clínica especializada em demência do Hospital de Clínicas de Porto Alegre (HCPA), Brasil, durante o período de junho de 2004 a junho de 2008. A avaliação dos pacientes consistiu de história médica, testes cognitivos, avaliação do estado funcional (Atividades de vida diária - AVD; atividades instrumentais de vida diária - AIVD) e Inventário Neuropsiquiátrico (INP) para avaliação comportamental. A gravidade da demência foi avaliada com a escala CDR. Todos os pacientes realizaram testes laboratoriais de rastreio e exames de imagem para definir a etiologia da demência.

**Resultados:**

Da amostra 71% eram mulheres; idade de 79±8 anos (média±DP) e escolaridade de 4±3 anos. Sessenta e quatro pacientes (60%) apresentavam diagnóstico de doença de Alzheimer; dentro da amostra 26.7% foram classificados como CDR=1; 44% como CDR=2 e 3% como CDR=3. Uma diferença significativa nos escores do MMSE e nos escores funcionais foi observada entre as categorias de CDR (gravidade). Não houve associação significativa entre gravidade e comprometimento nos testes de memória e sintomas comportamentais.

**Conclusões:**

Doença de Alzheimer foi a etiologia mais comum, e a seguir demência vascular. No diagnóstico, a maioria dos pacientes apresentou gravidade da demência de grau leve a moderado, independente da causa.

The aging of the Brazilian population is an ongoing reality, and currently the number of elderly people in Brazil is estimated at over 20 million.^1^ Among the major consequences of this growth is an increase in the prevalence of dementia, particularly Alzheimer's disease.^2,3^

Presently, dementia is thought to affect 24 million persons worldwide and is expected to rise to 42 million by 2020. Furthermore, 60% of this demented population lives in developing areas of the world, a proportion expected to increase to up to 71% by the year 2040.^4^

One of the Brazilian studies evaluating elderly people in the community found a prevalence of dementia of 7.1%.^3^ The same investigation also demonstrated that Alzheimer's disease was the most common etiology, followed by vascular dementia. Likewise, in most studies involving patients seen at outpatient dementia clinics, Alzheimer's disease was the most common type of dementia observed .^5-7^ Only one study showed a predominance of vascular dementia over Alzheimer's, albeit showing only a small difference between the diseases. The majority of studies also show a mean educational level of less than 5 years. Not all studies evaluated the severity of dementia at diagnosis. However, one study reported that almost 50% of cases were minimal or mild.^8^

Conditions that cause cognitive impairment, particularly dementias, are under diagnosed in the primary care setting.^9,10^ Patients may be not adequately evaluated, and the diagnosis ends up being delayed. A study which evaluated the rate of dementia in primary health care, observed that less than a half of the patients with dementia had their diagnosis documented in a primary medical care setting. This eventually leads to an increase in diagnoses of these cases in specialized hospital care settings.^11^

A few studies in Brazil have reported the different etiologies of dementia^3, 5-7,9,10^ but none of these were carried out in the South of the country.

The aim of this study was to evaluate the clinical and demographic profile of patients with dementing disorders seen at an outpatient clinic in a University Hospital in the city of Porto Alegre, Rio Grande do Sul State, investigating etiology, severity, cognitive and functional status, and behavioral symptoms.

## Methods

This study sample consisted of 105 demented patients seen at the Dementia Outpatient Clinic of Hospital de Clínicas de Porto Alegre (HCPA) during the period spanning from June 2004 to June 2008. The diagnosis of dementia was based on the Diagnostic and Statistical Manual of Mental Disorders - Fourth Edition (DSM-IV) criteria.^12^ The diagnosis of Alzheimer’s disease (possible or probable) was made according to the National Institute of Neurological and Communicative Disorders and Stroke - Alzheimer Disease and Related Disorders Association (NINCDS-ADRDA) criteria.^13^ The diagnosis of vascular dementia followed the National Institute of Neurological Diseases and Stroke - Association Internationale pour la Recherche et l'Enseignement en Neurosciences (NINDS-AIREN) criteria of probable and possible vascular dementia.^14^ The diagnosis of Frontotemporal dementia was based on the consensus on clinical diagnostic criteria for Frontotemporal lobar degeneration.^15^

Evaluation of patients entailed collection medical history and performing an exam and cognitive testing, which included the Mini Mental State Examination (MMSE),^16^ a word list (10-item list in a simple immediate recall paradigm - word span - WS)^17^ and Wechsler's immediate logical memory test (MLi).^18^ The Activities of Daily Living Scale (ADL) and the Instrumental Activities of Daily Living Scale (IADL)^19^ were used to evaluate functional status while the Neuropsychiatry Inventory (NPI) was employed to assess behavioral symptoms.^20^ The cutoff scores to indicate impairment on the word span was 4 ^17^ and for the logical memory test was 4.^18^

A laboratory evaluation was also performed and included complete blood count, serum sodium and potassium, urea, creatinine, glucose level, cholesterol, tryglycerides, alkaline phosphatase, γ-glutamyl transferase, transaminase concentrations, serum thyroxine, thyroid-stimulating hormone, serum VDRL, FTA-ABS, B12 vitamin, folic acid and computed tomography imaging of the head.

Patients were classified, according to dementia severity into mild, moderate and severe, based on the Clinical Dementia Rating Scale (CDR). ^21^

The study was approved by the Ethics Committee for Research of the Hospital de Clínicas de Porto Alegre. All subjects and/or their proxies signed an informed consent term.

### Data analysis

The statistical analysis was performed using the *Statistical Package for the Social Sciences* (SPSS for Windows 14.0) software. Descriptive statistics (mean, SD and frequency) were calculated for demographic data, symptoms of NPI, MMSE and CDR. Parametric data were analyzed by one-way ANOVA with Tukey’s post-hoc test. The Chi-square test (with Yates correction or Fisher exact) was used for the association analysis.

## Results

The frequency of type of dementia is shown in Table 1. Alzheimer's disease was the most frequent, followed by vascular dementia.

**Table 1:** Dementia type frequency.

	n	%
Probable Alzheimer	60	56.2
Possible Alzheimer	4	3.8
Vascular	19	18.1
Frontotemporal	1	1
Mixed	8	9.5
Parkinson's related	2	1.9
Undefined	10	9.5

Mean (±SD) age was 79±8 years, and mean (±SD) educational level was 4±3 years. Of the total sample, 71% were female. The scores on the MMSE ranged from 0 to 23 (mean=11; SD 5.5).

Of the whole sample, 26.7% were classified as mild dementia (CDR=1), 44% moderate (CDR=2) and 29. 3% severe (CDR=3).

When comparing patients with Alzheimer's and vascular dementia, these being the most frequent etiologies, a significant difference among the groups in relation to gender was detected, whereby the female gender predominated in AD, whereas males predominated in VD (Table 2).

**Table 2:** Comparison of demographic and clinical data between AD and VaD patients.

	Alzheimer	Vascular	p
Sex female (%)	74.6	47.4	0.027*
Age (mean±sd)	80.2±7.3	76.6±8.1	0.066**
Education	3.8 ±2.9	4.0±3.2	0.823**
MMSE	10.3±5.9	12.7±4.4	0.702**
NPI (f × g)	35±22.2	45.8±12.7	0.845**
NPI distress	14.6±8.8	13.3±11.2	0.828**
WS	1.9±1.6	2.2±1.3	0.488**
MLi	1.3±1.2	1.2±1.0	0.702**

* Chi Square;

** t Test.

We observed that 75% of Alzheimer's patients were classified as either CDR 2 or 3 (CDR 1=25%; CDR 2=39%; CDR 3=36%), whereas almost half of patients with vascular dementia were CDR 1 (CDR 1=46%; CDR 2=39 %; CDR 3=15%).

We also observed performances below the cutoff point in 90.3% of patients on the word span and in 98.5% on the immediate logical memory test (MLi). There was a significant difference in the scores for ADL, IADL and MMSE for dementia according to the CDR scale. Borderline significance (p=0.053) was observed for the WS test (one-way ANOVA with Tukey's *post hoc*) (Table 3).

**Table 3:** Variables associated with dementia severity.

	CDR 1 Mean (sd)	CDR 2 Mean (sd)	CDR 3Mean (sd)	F	p
MMSE	15.4 (4.2)^a,c^	10.6 (4.8)^b^	4.7 (3.8)	8.2	.000
NPI (f × g)	25.6 (18.6)	50.5(10.4)	30.0 (17.8)	18.2	.090
NPI distress	12.4 (9.5)	21.8(4.1)	13.2 (9.6)	0.65	.234
WS	2.3 (1.5)^c^	1.8 (1.4)	0.7 (1.3)	3.12	.053
MLi	1.6 (1.5)	1.2 (1.4)	0.7 (1.5)	25.6	.527
ADL	4.4 (3.3)^a,c^	7.5 (5.0)^b^	12.7 (4.5)	1.58	.000
IADL	8.9 (4.6)^c^	10.7(3.2)^b^	13.4 (1.8)	2.77	.001

* (f × g)=frequency and severity of neuropsychiatric behavioral symptoms;

** ANOVA F value;

a CDR1 vs CDR2 - p<0.05;

b CDR2 vs CDR3 - p<0.05;

c CDR1 vs CDR3 - p<0.05.

Frequency and severity of behavioral symptoms, as well as distress of the caregiver, showed no significant differences on the severity of dementia scale (one-way ANOVA with Tukey post hoc) (Table 3).

## Discussion

The present study evaluated the clinical and demographic characteristics of dementia patients from an outpatient clinic seen during a period of 4 years.

Alzheimer's disease was the most frequent cause, with patients being predominantly at moderate to severe stages according to CDR, at time of diagnosis. A clear predominance of females was also observed. These patients showed lower educational attainment.

After Alzheimer's disease, vascular dementia was the second most frequent cause of dementia. This finding is similar to results observed in most studies carried out in Brazil^3,5-7^ and in outpatient clinics from tertiary facilities. Only one report showed vascular dementia and males as the most prevalent aspects of the sample.^8^

The frequent co-morbidities and the characteristics of the health service - a reference center for the diagnosis and treatment of dementia - could explain the high rate of unspecified dementia. Furthermore, the majority of patients were at a severe stage of the disease on the first evaluation where this fact could have precluded a better approach and hampered evaluation of important symptoms for diagnosis.

The mean educational level of the subjects in this sample (4 years) was similar to the average for the elderly population of Rio Grande do Sul state (4.1 years), but was lower than that observed in the city of Porto Alegre (7.1 years),^1^ home to 90% of the study participants. One explanation for the lower educational level of these patients in relation to the elderly population of Porto Alegre could be the association with higher prevalence of dementia observed in previous investigations.^22-25^

At diagnosis, 71.8% of patients were at a moderate to severe stage of the disease, reflecting the difficulty in performing early diagnosis in this facility. This may in part be due to lack of awareness of the population regarding symptoms of dementia and the general assumptions of memory problems during normal aging. On the other hand, the use of cognitive screening in the routine evaluation of elderly people with cognitive problems is not a general rule in the medical practice. Thus, specialized medical care is only sought when symptoms begin to exert an evident functional impact and other neuropsychiatric symptoms, when the disease is already at more advanced stage.^6,11^

The scores on the MMSE presented a decline across the three categories of severity (CDR 1, 2 and 3). We recognized congruence between the direction of the decline of the MMSE scores and worsening severity. However, the average scores on the MMSE in each category were considered low for the corresponding CDR. Nevertheless, level of education could be an important factor for this finding. Similarly, worsening on functional scores (Activities of Daily Living and Instrumentals Activities of Daily Living) was observed. These functional instruments of assessment are important for following up dementia patients, as well as measuring deterioration and guiding appropriate management.

Neuropsychiatric symptoms are commonly found in elderly patients with dementia and AD. The relationship between the prevalence of neuropsychiatric symptoms and the severity of dementia has varied widely in several studies, and may not present a significant difference^26^ in our sample. The frequency and severity of behavioral symptoms, as well as family distress did not increase with severity. Frequency of neuropsychiatric symptoms and distress was higher among patients at a moderate stage of dementia, independent of diagnosis.

Studies such as these, as well as epidemiological investigations, are very important to raise awareness of the proportion of different etiologies of dementia in Brazil, enabling the development of more specific prevention strategies and early diagnosis.
